# Survival in pregnancy-associated breast cancer patients compared to non-pregnant controls

**DOI:** 10.1186/s12958-024-01206-w

**Published:** 2024-03-26

**Authors:** María Martín Cameán, Ibon Jaunarena, Jose Ignacio Sánchez-Méndez, Covadonga Martín, Félix Boria, Elena Martín, Emanuela Spagnolo, Ignacio Zapardiel, Alicia Hernández Gutiérrez

**Affiliations:** 1 Department of Gynecology and Obstetrics, La Paz University Hospital, Madrid, 28046 Spain; 2 Gynecologic Oncology Unit, Department of Gynecology and Obstetrics, Donostia University Hospital, San Sebastian, 20014 Spain; 3 Clínica Universidad de Navarra, Madrid, 28027 Spain

**Keywords:** Breast cancer, Pregnancy, Chemotherapy, Neonatal outcome

## Abstract

**Background:**

Pregnancy-associated breast cancer (PABC) is a rare entity whose prognosis has previously been studied and is subject to controversy.

**Methods:**

Survival of patients with PABC diagnosed between 2009 and 2021 with breast cancer during pregnancy or until 1 year after childbirth was compared with non-pregnant patients with breast cancer from the same period at La Paz University Hospital. Cox proportional hazards regression was used to compare disease-free (DFS) and overall (OS) survival between the groups, adjusting for grade and pathologic stage.

**Results:**

Among the 89 included patients with breast cancer, 34 were diagnosed during pregnancy, and 55 were not pregnant. The pregnant patients were more likely to have grade 3 tumors (61.3% vs 37%, *p* = 0.023) and an advanced stage (pathologic stage III-IV: 44.1% vs 17.6%, *p* = 0.008). Median follow-up was 47 months for the pregnant group and 46 months for the control group. After adjustments for tumor grade and pathologic stage, OS was comparable between the groups (HR 2.03; 95% CI 0.61 to 6.79; *P* = 0.25).

**Conclusions:**

The outcome of women diagnosed with PABC is comparable to young non-pregnant controls. However, it should be taken into account that PABC has a more aggressive phenotype.

## Introduction

The number of women diagnosed with breast cancer during pregnancy has increased recently, in part due to delaying motherhood to older ages [1, 2]. The treatment of these patients is challenging and requires careful balancing between the treatment of the mother and the safety of the fetus [1, 3, 4].

Cancer during pregnancy is a rare entity, with an approximate incidence of 1:1000 pregnancies [5]. Breast cancer is the most frequent type, followed by cervical cancer, lymphoma, ovarian cancer, leukemia, colorectal cancer, and melanoma [1, 6]. Pregnancy-associated breast cancer (PABC) has been reported to affect 1 in 3000–10,000 pregnancies worldwide, corresponding to approximately 2000–4000 new cases in Europe annually [1].

Pregnancy appears to have 2 opposing effects on the maternal risk of developing breast cancer: a temporal increasing risk during the first years after pregnancy, followed by a long-term protective effect. In fact, PABC is defined as a case that occurs during pregnancy or within a year afterward [1, 2, 7, 8]. However, more recently, it has been proposed to consider cancer that occurs during pregnancy as a different entity from cancer diagnosed in the postpartum period, which, according to evolving evidence, can extend up to 5–15 years after delivery and which appears to have poorer prognosis [1, 9–11].

Given that the safety of treatment during pregnancy has been demonstrated in recent years, the number of patients receiving treatment during pregnancy is increasing [12]. This change has led to fewer pregnancy interruptions and fewer induced premature births. Possible risks are attenuated as pregnancy progresses. Most guidelines recommend chemotherapy treatment during the second and third trimesters, whereas radiotherapy, hormonal, and biological treatments should be delayed until after delivery [1, 13]. PABC prognosis can be affected by various factors, such as delays in diagnosis and treatment, tumor growth stimulation from pregnancy hormones, and associated immunosuppression [1].

There is evidence suggesting that breast cancer diagnosed during pregnancy and around delivery exhibits more aggressive behavior, including a higher tumor grade, larger size, negative estrogen and progesterone receptor status, HER2 expression, lymphovascular invasion, and lymphocytic infiltration [1, 2, 11].

The physiologic gestational changes, such as the increase in plasma volume or glomerular infiltration rate, cause decreased drug concentrations with a potential impact on chemotherapeutic efficacy. There is some disagreement about PABC prognosis, although the latest evidence has shown that the survival of those who received chemotherapy during pregnancy is comparable with non-pregnancy-associated breast cancer [9, 14].

PABC prognosis has previously been addressed in several studies [2, 5, 8, 9, 13–16], with considerable controversy around this issue. Some have observed poorer survival among women with PABC [2, 13, 15, 17]. The aim of this study was to estimate the impact of pregnancy on breast cancer prognosis, analyzing the results of a national reference center in order to contribute data to the literature as well as to provide detailed information to patients who will be treated at our center.

## Materials and methods

### Study design

This was a single-center retrospective case–control study*.* The study group included patients diagnosed with breast cancer during pregnancy or until 1 year after childbirth, from 2009 to 2021, at La Paz University Hospital, Madrid. Data collected were independent of the outcome of the pregnancy, cancer stage, or treatment received. The control group included young patients (≤ 40 years) diagnosed with breast cancer without pregnancy 5 years prior to diagnosis at La Paz University Hospital from 2009 to 2021.

This study was approved by the La Paz University Hospital ethics committee (version 4.3, 15 Jun 2018, HULP PI-3930). Informed consent was not required due to the retrospective nature of the study, according to ethics committee approval.

All patient information was determined by reviewing computerized medical histories. We collected information about demographic data, date of diagnosis, tumor characteristics, surgery, chemotherapy, radiotherapy, endocrine and biologic treatment, and patient outcomes. In the pregnant group, information about the pregnancy was also collected: last menstrual period, type of conception and pregnancy, maternal and fetal complications, treatment received, delivery, newborn weight, arterial pH, Apgar test, and child outcome.

All patients included in the study were diagnosed and treated according to the hospital’s protocols and were staged according to the American Joint Committee on Cancer staging system (seventh edition) [18]. Various imaging techniques were used for the diagnosis: ultrasound, mammography, magnetic resonance, and core needle biopsy. All cases were presented to a multidisciplinary committee composed of gynecologists, oncologists, radiotherapists, surgeons, and pathologists.

The primary outcome was the difference in overall survival (OS) and disease-free survival (DFS) between patients diagnosed with PABC and patients not diagnosed during pregnancy. OS was defined as time (in months) from cancer diagnosis to death, and disease-free survival (DFS) was defined as time (in months) from cancer diagnosis to locoregional or distant recurrence of disease.

The secondary outcome was newborn outcome. Obstetrical and neonatal data were collected: type of conception, complications during pregnancy, congenital malformations, delivery data, gestational age at birth, Apgar test, birth weight, and sex.

### Statistical analysis

Qualitative data were described as absolute frequencies and percentages. Quantitative data were described as mean and standard deviation (SD) if they followed a normal distribution; variables that did not follow a normal distribution were defined as median and interquartile range.

The normality of the continuous variables was studied using the Kolmogorov–Smirnov test. The chi-squared test or Fisher’s exact test was employed to study the association between categorical variables, and Student’s t-test for the association between quantitative variables.

The analyses of survival and disease-free time were performed with a Kaplan–Meier analysis calculated from the date of diagnosis to the date of death or recurrence. To compare survival functions by group, log-rank tests were performed.

Multivariate Cox proportional hazard models were used to estimate hazard ratios (HRs) and 95% confidence intervals (CIs) of the association between pregnancy and OS. The confounding factors under consideration were tumor grade and pathologic stage.

All statistical tests were considered bilateral, and *P* values less than 0.05 were considered significant. The data were analyzed with the IBM SPSS Statistics version 29.0. software package (SPSS, Chicago, IL, USA).

## Results

**Fig. 1:**
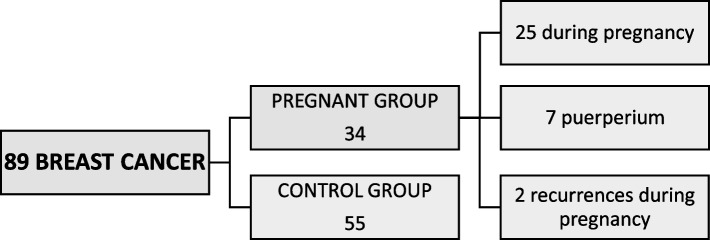
Patients included on the study

Among the 89 patients with breast cancer, 34 (38.2%) were diagnosed during pregnancy. Of these, 25 patients were diagnosed with primary breast cancer during any trimester of pregnancy. Seven patients were diagnosed with primary breast cancer within the first year after delivery. In 2 patients, the diagnosis was a recurrence of breast cancer during gestation; one of them was a recurrence in the contralateral breast of a breast cancer she had 4 years earlier, and the other was a lymph node recurrence of a cancer diagnosed one-and-a-half years earlier (Fig. 1).

The control group included 55 patients diagnosed with breast cancer without any association with pregnancy. All of the patients were younger than 40 years of age at the time of diagnosis (ratio pregnant/nonpregnant 1:1.62). Demographic features are summarized in Table 1.

**Table 1:** Demographics variables

Variable	Non pregnant group (*N* = 55)		Pregnant group (*N* = 34)		*P*-value	Missing values			
						Nonpregnant		Pregnant	
	No	%	No	%		No	%	No	%
**Delivery**					0.049	21	38.2	3	8.8
0	18	52.9	9	33.3					
1	7	20.6	15	48.4					
≥ 2	9	26.5	7	22.6					
**Smoker**					0.003	36	65.5	6	17.6
No	9	52.9	26	92.9					
Yes	8	47.1	2	7.1					
**BRCA**					0.157	39	70.9	18	52.9
Normal	6	37.5	10	50					
Mutation	10	62.5	6	37.5					
**Hormonal contraceptive**					0.157	37	67.3	20	58.8
No	3	16,7	7	50					
Yes	15	62,5	7	50					

Median age was 34.88 years (± 4.32) for the pregnant group and 33.58 (± 4.03) for the control group. The most common histological type in both groups was invasive ductal carcinoma (97.1% in PABC and 85.5% in the control group). Clinicopathologic variables are depicted in Table 2.

**Table 2:** Clinicopathologic variables

Variable	Non-pregnant (*N* = 55)		Pregnant (*N* = 34)		*P*-value	Missing values			
						Non-pregnant		Pregnant	
	No	%	No	%		No	%	No	%
**Pathology**					0.369	3	5.5	4	11.8
Unifocal	41	78.8	21	70					
Multifocal	11	21.2	9	30					
**Malignancy Grade**					**0.023**	1	1.8	3	8.8
1	8	14.8	0						
2	26	48.1	12	38.7					
3	20	37	19	61.3					
**Molecular**					0.148	3	5.5	2	5.9
ER/PR + , Ki67 < 20	13	25	2	6.3					
ER/PR + , Ki67 ≥ 20	14	26.9	9	28.1					
ER/PR + , Ki67 ≥ 20,Her2 +	10	19.2	10	50					
Her2 + (ER/PR-)	6	11.5	2	6.3					
Triple Negative (ER/PR/HER2 negative)	9	17.3	9	28.1					
**Pathologic stage (Grouped)**					**0.008**	4	7.3	0	
0,I,II	42	82.4	19	55.9					
III,IV	9	17.6	15	44.1					

*Abbreviations: ER* Estrogen receptor, *PR* Progesterone receptor, *ER/PR* + ER and/or PR positive (≥ 1%), *HER2* Human epidermal growth factor receptor2

### Treatment

The type of oncological treatment received is described in Table 3. Within the PABC group, 8 (25.8%) patients underwent surgery during pregnancy, the majority (50%) of them during the second trimester, 2 in the first, and the other 2 in the third trimester. A total of 16 (48.5%) patients received chemotherapy during pregnancy. The chemotherapy schedule most administered (87.5%) during pregnancy was a combination of an anthracycline (epirubicin) + alkylating agents (cyclophosphamide). It was not until after delivery that taxanes were administered. Among the control group, the chemotherapy regimen most prescribed was anthracycline (epirubicin) + alkylating agents (cyclophosphamide) + 5-fluorouracil + taxanes. Regimens were administered at standard of care dosages.

**Table 3:** Oncological treatment

Variable	Non-pregnant group (*N* = 55)		Pregnant group (*N* = 34)		*P*-value	Missing values			
						Non-pregnant		Pregnant	
	No	%	No	%		No	%	No	%
**Breast surgery**					0.187	0		1	2.9
None	0	0	2	6.1					
Lumpectomy	30	54.5	13	39.4					
Mastectomy	20	36.4	14	42.4					
Bilateral mastectomy	5	9.1	3	9.1					
Quadrantectomy	0	0	1	3					
**Lymph node surgery**					0.487	0		3	8.8
None	1	1.8	2	6.5					
SLNB^a^	36	65.5	18	58.1					
ALND^a^	18	32.7	11	35.5					
**Chemotherapy setting**					0.070	1	1.8	0	
None	9	16.7	2	5.9					
Neoadjuvant	35	64.8	19	55.9					
Adjuvant	10	18.5	13	38.2					
**Radiotherapy**					0.189	0		0	
None	11	20	11	32.4					
Yes	44	80	23	67.6					
**Endocrine Therapy**					0.692	1	1.8	4	11.8
None	15	27.8	11	36.7					
Tamoxifen	28	51.9	14	46.7					
Aromatase inhibitor	11	20.4	5	16.7					

^a^*Abbreviations: SLNB* Sentinel lymph node biopsy, *ALND* axillary lymph node dissection

### Survival

**Fig. 2:**
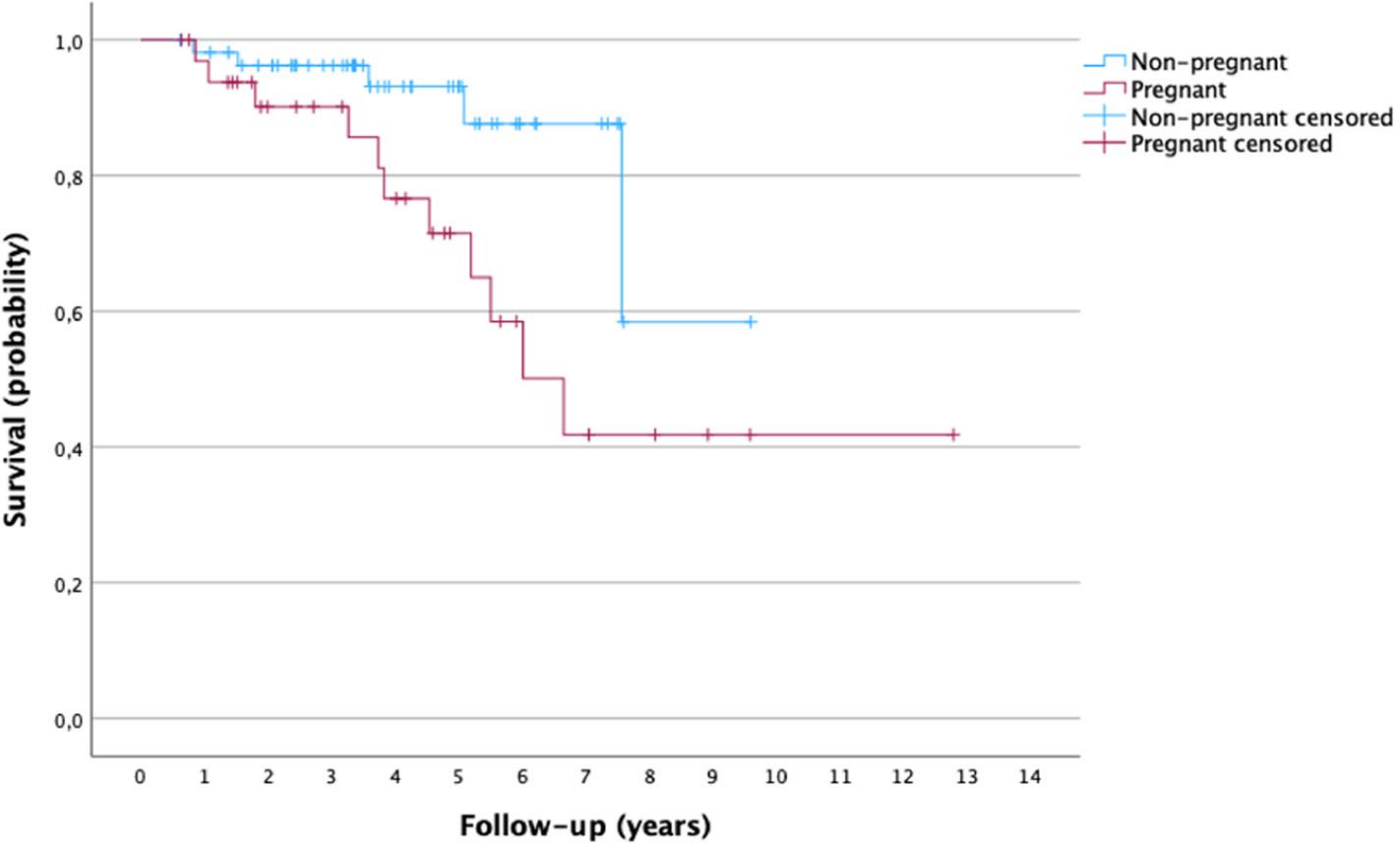
Kaplan–Meier curves for Overall Survival (OS) for the pregnant and nonpregnant groups. Log rank: 0.02

Median follow-up was 47 months for the pregnant group and 46 months for the control group. During follow-up, 11 (33.3%) patients in the PABC group and 5 (9.1%) in the control group died. The observed 5-year OS for the pregnant and control group was 65.0% and 87.6%, respectively. The median survival time was 6 years for the study group and 7 years for the control group (Fig. 2).

**Fig. 3:**
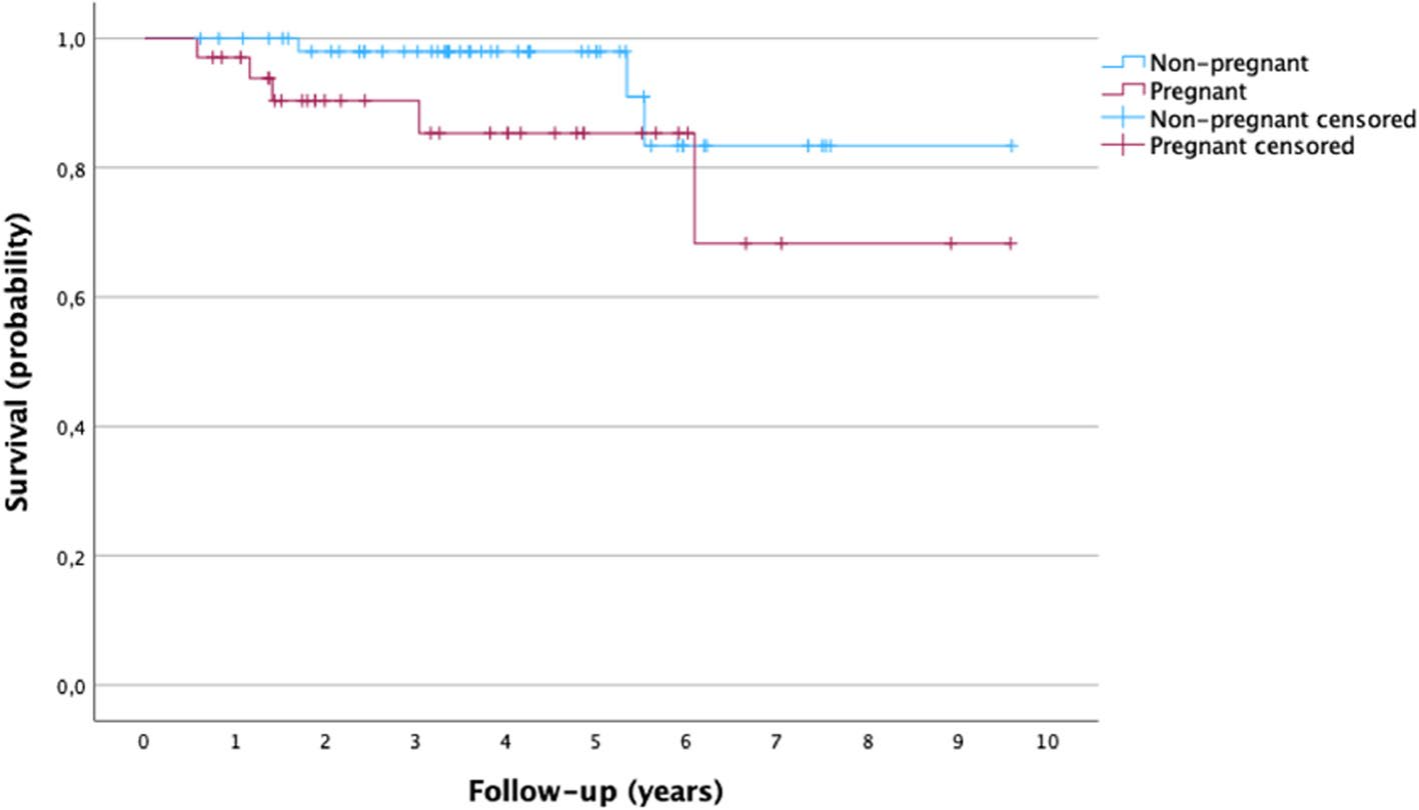
Kaplan–Meier curves for Disease Free Survival (DFS) for the pregnant and nonpregnant groups. Log rank:0.13

Figure 3 shows the results obtained for the observed 5-year DFS for both groups; 85.3% for the pregnant group and 91% for the nonpregnant group. These differences were not statistically significant (log rank: 0.13).

After adjustments for tumor grade and pathologic stage, we found no evidence of poorer prognosis for women diagnosed with PABC regarding OS (HR 2.03; 95% CI 0.61 to 6.79; *P* = 0.25).

### Obstetrics and neonatal outcome

Data from 34 pregnancies were collected. Data from 2 were excluded: one was a first-trimester miscarriage and the other was a terminated pregnancy. Two of the remaining 32 pregnancies were twins, both of them dichorionic diamniotic. Thirty (93.8%) pregnancies were naturally conceived, and 2 (6.3%) by in vitro fertilization. Chemotherapy was administered during 15 (45.4%) pregnancies, and 8 (24.2%) patients underwent surgery during pregnancy. Most (82.1%) of the pregnant women did not suffer any complication. Minor complications were reported: 1 diet-controlled gestational diabetes, 1 preeclampsia, 1 small for gestational age, 1 abnormal placentation (placenta previa), and 1 threatened preterm labor (which ended in full-term delivery). Only 2 patients received any treatment different from the oncologic one: tocolytic treatment and prophylactic acetylsalicylic acid. Eight (29.6%) patients had a spontaneous delivery. Eleven (40.7%) deliveries were induced, 75% of which were due to oncologic causes (labor induction in order to start chemotherapy drugs that are not recommended during pregnancy). Cesarean section was scheduled for 8 (29.6%) patients. Most (51.9%) had a vaginal delivery, 44.4% ended in cesarean section, and there was only 1 instrumental delivery (vacuum).

A total of 34 children (including 2 pairs of twins) were born from 32 mothers diagnosed with PABC. Average birth weight was 2819 gr (± 488). The number and type of congenital malformations were similar to those in the general population [19, 20]; 1 was diagnosed with bilateral cryptorchidism and 1 with testicular hydrocele.

## Discussion

The most remarkable finding from this study is the difference in prognosis between the groups: 33.3% died in the pregnant group vs 9.1% in the control group, with a statistically significant shorter OS in the pregnant group. However, after adjustment for tumor characteristics (grade and pathologic stage), these associations were attenuated. These findings support previous studies [2, 5, 11, 13, 14, 16] that suggest pregnancy-related breast cancer is more likely to present with a larger tumor, a higher stage, more poorly differentiated, and with a higher frequency of lymph node metastases. In agreement with the literature, in this study we found different tumor characteristics when comparing the groups, with PABC showing a more aggressive profile. In the pregnant group there was a higher prevalence of triple-negative tumor, poorer differentiation, and diagnosis in more advanced stages compared with the non-pregnant group.

Several explanations have been proposed for the poorer prognosis of pregnancy-related breast cancer. One is delayed diagnosis, which can occur in pregnant women because palpable masses or lumps can be ascribed to normal breast changes during pregnancy. To prevent this, any mass or lump during pregnancy or postpartum should warrant further work up.

The mechanisms driving PABC remain unclear, and many hypotheses have been postulated. During pregnancy, elevated levels of estrogen, progesterone, and insulin growth factor induce breast cell proliferation and could initiate tumorigenesis or stimulate growth of cells that have already undergone malignant transformation. However, the majority of PABC does not express hormonal receptors. In xenograft models of PABC it has been shown that, although lacking hormonal expression, systemic estrogens are needed for their formation and the progression of ER-negative cancers [11]. Another factor that influences the development of PABC is the combination of immunosuppression associated with pregnancy, immune tolerance, and inflammatory changes related to mammary gland involution [9–11].

Due to the poorer PABC prognosis, an increased awareness among clinicians and patients could help achieve earlier diagnoses. We suggest including clinical breast exam as part of the pregnancy visit protocol and request imaging test if there is any suspicion of breast pathology. Previous studies have suggested that ultrasound and mammography with fetal shielding are both appropriate diagnostic tools with no fetal risk [9, 16].

PABC prognosis has previously been addressed in several studies, with considerable controversy around this issue. A meta-analysis including 3628 cases and 37,100 controls showed a significantly higher risk of death in PABC compared with those not pregnant (pHR: 1.44; 95% CI [1.27–1.63]). However, this difference was only statistically significant in the group diagnosed postpartum (pHR: 1.84; 95% CI [1.28–2.65]) and not in the group diagnosed during pregnancy (pHR: 1.29; 95% CI [0.74–2.24]) [15]. The study by Viuff et al. [13] found higher overall mortality among women with PABC in the first 2 years after diagnosis compared with non-pregnant women with breast cancer, including 156 and 11,110 patients, respectively (HR 2.28 [1.48–3.52)]. In contrast, survival was comparable between the groups from 2 years after diagnosis.

A later study by Amant et al. [14], which included 311 women with PABC compared with 865 women with breast cancer who were not pregnant, found similar OS in both groups. Nevertheless, regarding the DFS analysis, although the HR 1.34 result (95% CI; 0.93–1.91) suggests better outcomes for the nonpregnant group, it is not statistically significant*.* An extension of this study focused on a subgroup of patients who received chemotherapy during pregnancy, comparing the prognosis between PABC treated with standard chemotherapy regimens and a non-pregnant control group [9]. Although comparable results were reported in terms of DFS and OS, a poorer OS was suggested for pregnant women receiving chemotherapy for luminal A breast cancer.

PABC treatment is challenging for clinicians, patients, and their families, because it requires careful balancing between the treatment of the mother and the safety of the fetus. Guidelines recommend following the same treatment that is standard for non-pregnant patients. Following this recommendation, no differences were observed in our study between the study and control groups in the treatment received, the only exception being that taxane-based chemotherapy and radiotherapy were delayed until birth, following the standard recommendations at the time of these patients’ diagnosis. Breast cancer surgery can be performed safely during any stage of pregnancy [1]. Chemotherapy, in a neoadjuvant or adjuvant setting, including anthracyclines, fluoropyrimidines, taxanes, and platinum derivates, is feasible after 12 weeks of pregnancy [1, 5, 21]. However, hormonal therapy is not recommended during pregnancy and should be delayed until birth [22], and trastuzumab is also contraindicated during pregnancy [23]. Radiotherapy is possible during the first half of pregnancy [1].

Despite prenatal exposure to maternal cancer, the associated maternal distress, diagnostic procedures, and oncological treatment including chemotherapy, the outcome for children in our study did not differ from that of the general population, in line with previous literature. Normal behavioral competence and cognitive and cardiac outcomes have been reported in children up to 18 years of age for fetuses exposed to chemotherapy in utero [24–27].

There are some limitations to this study that must be taken into account. This was a single-center study which, added to the rarity of this entity, resulted in a small sample size; therefore, it achieved insufficient power in the statistical analysis. Also, due to small size of the groups, it was not possible to analyze the results by the subgroup of patients diagnosed during pregnancy and during the postpartum period.

## Conclusions

In our cohort of patients, we found poorer survival in those diagnosed with breast cancer during pregnancy or at 1 year postpartum when compared with nonpregnant patients with breast cancer, as a consequence of a more aggressive PABC phenotype. The group with PABC also had a more advanced stage and the cancer was more poorly differentiated than in controls. Our data show the greatest observed difference in prognosis and tumor characteristics between the two groups. After adjustment for tumor characteristics, the difference in survival disappeared. Increased awareness among patients and specialists could help achieve earlier diagnoses. Among patients with PABC, no higher rate of obstetric or neonatal complication was identified than that in the general population. 

## Data Availability

No datasets were generated or analysed during the current study.
