# Correction: Survival in pregnancy-associated breast cancer patients compared to non-pregnant controls

**DOI:** 10.1186/s12958-024-01244-4

**Published:** 2024-06-15

**Authors:** María Martín Cameán, Ibon Jaunarena, Jose Ignacio Sánchez-Méndez, Covadonga Martí, Félix Boria, Elena Martín, Emanuela Spagnolo, Ignacio Zapardiel, Alicia Hernández Gutiérrez

**Affiliations:** 1 Department of Gynecology and Obstetrics, La Paz University Hospital, Madrid, 28046 Spain; 2 Gynecologic Oncology Unit, Department of Gynecology and Obstetrics, Donostia University Hospital, San Sebastian, 20014 Spain; 3 Cl?nica Universidad de Navarra, Madrid, 28027 Spain

**Correction: Reprod Biol Endocrinol 22, 34 (2024)**

10.1186/s12958-024-01206-w

Following publication of the original article [1], the authors reported an error to the presentation of names in the author group.

The incorrect author names are:

Author 1: Given name: María

Given name: Martín

Last name: Cameán

Author 2: Given name: Ibon

Last name: Jaunarena Marin

Author 3: Given name: Jose

Given name: Ignacio

Given name: Sánchez

Last name: Mendez

Author 4: Given name: Covadonga

Last name: Martí Alvarez

Author 5: Given name: Félix

Last name: Boria Alegre

Author 6: Given name: Elena

Last name: Martín Boado

The correct author names are:

Author 1: Given name: María

Last name: Martín Cameán

Author name 2: Given name: Ibon

Last name: Jaunarena

Author name 3: Given name: Jose

Given name: Ignacio

Last name: Sánchez-Méndez

Author name 4: Given name: Covadonga

Last name: Martí

Author name 5: Given name: Félix

Last name: Boria

Author name 6: Given name: Elena

Last name: Martín

The original article [1] has been updated.
